# Dynamic Luminal Topography: A Potential Strategy to Prevent Vascular Graft Thrombosis

**DOI:** 10.3389/fbioe.2020.573400

**Published:** 2020-08-31

**Authors:** Nandan N. Nath, Luka Pocivavsek, Joseph A. Pugar, Ya Gao, Karim Salem, Nandan Pitre, Ryan McEnaney, Sachin Velankar, Edith Tzeng

**Affiliations:** ^1^Division of Vascular Surgery, University of Pittsburgh Medical Center, Pittsburgh, PA, United States; ^2^Division of Vascular Surgery, The University of Chicago, Chicago, IL, United States; ^3^Department of Chemical Engineering, University of Pittsburgh, Pittsburgh, PA, United States; ^4^Department of Bioengineering, University of Pittsburgh, Pittsburgh, PA, United States; ^5^VA Pittsburgh Healthcare Systems, Pittsburgh, PA, United States; ^6^Department of Mechanical Engineering and Materials Science, University of Pittsburgh, Pittsburgh, PA, United States

**Keywords:** prosthetic vascular graft, dynamic topography, compliance, platelets, *ex vivo* pulsatile flow model

## Abstract

**Aim:**

Biologic interfaces play important roles in tissue function. The vascular lumen-blood interface represents a surface where dynamic interactions between the endothelium and circulating blood cells are critical in preventing thrombosis. The arterial lumen possesses a uniform wrinkled surface determined by the underlying internal elastic lamina. The function of this structure is not known, but computational analyses of artificial surfaces with dynamic topography, oscillating between smooth and wrinkled configurations, support the ability of this surface structure to shed adherent material (Genzer and Groenewold, 2006; Bixler and Bhushan, 2012; Li et al., 2014). We hypothesized that incorporating a luminal surface capable of cyclical wrinkling/flattening during the cardiac cycle into vascular graft technology may represent a novel mechanism of resisting platelet adhesion and thrombosis.

**Methods and Results:**

Bilayer silicone grafts possessing luminal corrugations that cyclically wrinkle and flatten during pulsatile flow were fabricated based on material strain mismatch. When placed into a pulsatile flow circuit with activated platelets, these grafts exhibited significantly reduced platelet deposition compared to grafts with smooth luminal surfaces. Constrained wrinkled grafts with static topography during pulsatile flow were more susceptible to platelet accumulation than dynamic wrinkled grafts and behaved similar to the smooth grafts under pulsatile flow. Wrinkled grafts under continuous flow conditions also exhibited marked increases in platelet accumulation.

**Conclusion:**

These findings provide evidence that grafts with dynamic luminal topography resist platelet accumulation and support the application of this structure in vascular graft technology to improve the performance of prosthetic grafts. They also suggest that this corrugated structure in arteries may represent an inherent, self-cleaning mechanism in the vasculature.

## Introduction

The tissue-blood interface represents a critical, dynamic interaction between endothelial cells (ECs) and circulating red blood cells (RBCs), platelets, and inflammatory cells (Ignarro et al., 1987; Palmer et al., 1987; Patel et al., 2003; Dubois et al., 2007; Furie and Furie, 2008; Bennett et al., 2016) to prevent cell adherence and thrombosis. While ECs and underlying smooth muscle cells are vital to vascular homeostasis and patency (Ignarro et al., 1987; Bennett et al., 2016), the contribution of macroscopic arterial luminal surface geometry to the mechanical prevention of thrombus formation has not been previously evaluated. Natural surfaces such as the luminal lining of arteries are superior at resisting thrombosis or unwanted fouling compared to artificial surfaces (Allen, 1969; Russell, 2002; Genzer and Groenewold, 2006; Bixler and Bhushan, 2012; Li et al., 2014). Medical research has heavily focused on improving the antithrombotic properties of synthetic surfaces exposed to blood such as in catheters, dialysis circuits, vascular grafts, and heart valves (Weber et al., 2002; Jordan and Chaikof, 2007; Lavery et al., 2017). Attempts to seed ECs on prosthetic grafts have been largely unsuccessful or not feasible (Seifalian et al., 2002). One popular strategy is surface modification through heparin bonding (Hasebe et al., 2006; Biran and Pond, 2017). However, studies have shown only limited benefit of this approach in improving graft patency (Devine et al., 2004; Bosiers et al., 2006; Uhl et al., 2017). Currently, commonly used synthetic vascular grafts are manufactured from polytetrafluorethylene (PTFE) and polyethylene terephthalate (PET). While PTFE and PET function extremely well in large caliber vascular reconstruction, both have inferior patency compared to autologous vein and perform poorly in smaller caliber arterial reconstruction (Quinones-Baldrich et al., 1992; Albers et al., 2005; Takagi et al., 2010; Twine and McLain, 2010; Neville et al., 2012; Uhl et al., 2017). Yet, these materials have been in existence for over half a century without significant modification (Naoum and Elias, 2012). The development of innovative approaches to antithrombosis in vascular graft design is an ongoing challenge.

**FIGURE 1:**
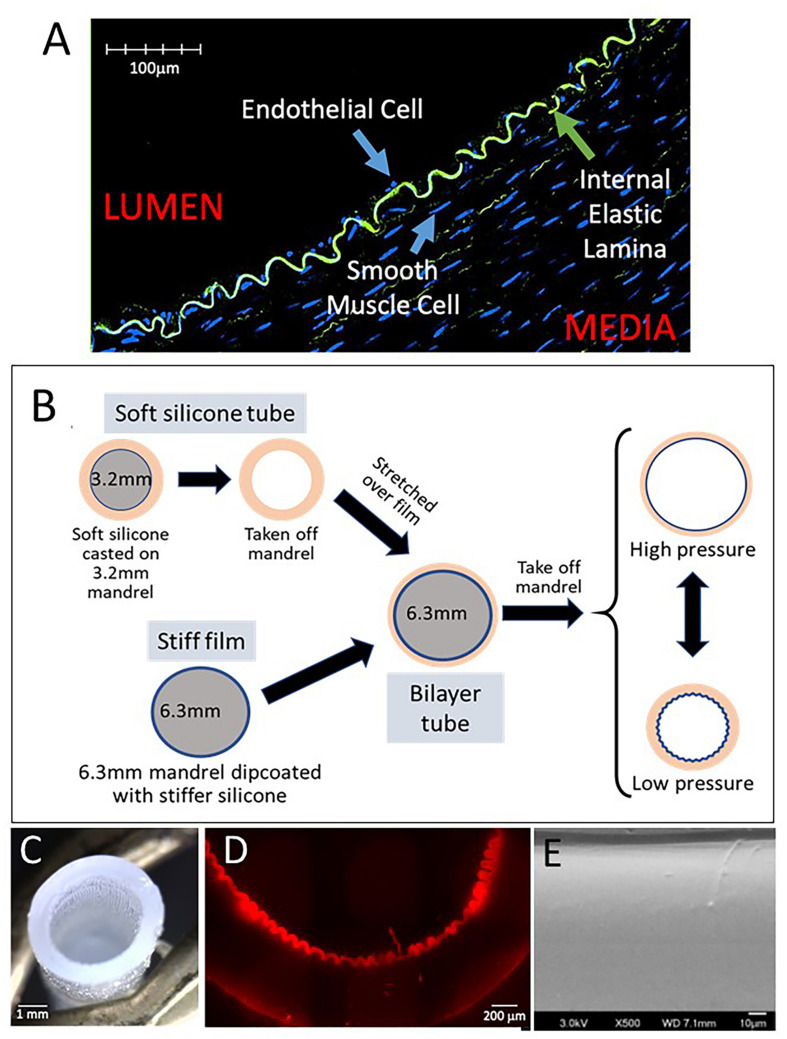
Fabrication of dynamic silicone wrinkled graft. **(A)** Representative confocal image of a cross-section of pig carotid artery fixed under luminal pressure of 60 mmHg demonstrates the wrinkled configuration of the internal elastic lamina (green autofluorescence for elastin), resulting in a wrinkled luminal surface lined by endothelial cells. **(B)** Schematic representation of the fabrication of a bilayer silicone graft with a wrinkled inner lining under resting conditions. A thin film composed of a stiffer silicone is dip-coated on a 6.3 mm mandrel. An outer tube composed of a soft silicone is casted on a 3.2 mm mandrel. This soft tube is removed from the mandrel and stretched over the stiff film on the 6.3 mm mandrel. Removal of this bilayer graft from the larger mandrel results in recoil of the outer tube. The stiffer inner film buckles into a uniform wrinkled luminal surface. This bilayer graft can distend with pressure which results in flattening of the luminal surface. Under lower pressures, the graft relaxes and the luminal wrinkles reform. **(C)** Photograph of the bilayer graft showing luminal corrugations. **(D)** Representative photomicrograph of a cross section of the graft showing the wrinkled inner film. **(E)** Representative scanning electron microscopy of the luminal surface of wrinkled grafts showing the smooth characteristics of the silicone surface without any nanoscale topography.

In nature, there are many examples of surfaces that are at risk of accumulating debris. A closer look at these surfaces often reveals a non-flat topography with a consistent wrinkling pattern or continuous surface undulation (Cerda, 2005; Bhushan, 2009; Pocivavsek et al., 2009; Yang et al., 2010; Shivapooja et al., 2013). Biological structure is often linked to function and suggests that surface topography may serve a critical role in antifouling of a system. In prior studies, nanoscale static topography with high density structure, related to the material composition, has been shown to reduce platelet adhesion and activation as well as support endothelial growth and alignment (Koh et al., 2010; Uttayarat et al., 2010; Xu and Siedlecki, 2017). In contrast, Pocivavsek et al. (2008, 2018, 2019) reported a biophysical analysis of a possible novel mechanism of surface cleansing in nature and in innovation, namely that of a dynamic “wrinkled surface” or dynamic macro-topography. We define dynamic topography as reversible changes in the surface landscape of a material, cycling between wrinkled and smooth configurations. Pocivavsek et al. (2018) showed that surfaces with dynamic topography can force deadhesion of soft films adherent to the surface and developed an energy-based delamination model to further evaluate this function. Interestingly, a similar wrinkled topography exists on the luminal surface of human and animal arteries (Svendsen and Tindall, 1988) (Figure 1A), suggesting that macro-scale topographical structure may serve an anti-fouling function in nature.

The ability of dynamic topography to reduce platelet aggregation on a synthetic surface has been demonstrated (Pocivavsek et al., 2019). Planar as well as tubular surfaces were constructed to reversibly transition between flat and wrinkled states. When exposed to whole blood, these dynamic surfaces showed much lower blood cell adherence compared to static surfaces with the same surface chemistry and nano-structure (Pocivavsek et al., 2019). This was also true for both hydrophilic and hydrophobic surfaces, suggesting that the anti-fouling activity is mechanical and independent of surface chemistry. In this current study, we hypothesize that we can fabricate compliant prosthetic grafts that exhibit dynamic luminal topography and are more resistant to platelet aggregation than grafts lacking this topography.

## Methods

### Graft Fabrication and Compliance Testing

Compliant vascular grafts capable of dynamic topography were fabricated as illustrated (Figure 1B). A blend of soft silicone elastomers (MG7-9900, MDX4-4210, Dow Chemicals, Midland, MI, United States) was molded onto a 3.2 mm diameter mandrel, producing an elastomer tube with a wall thickness of approximately 0.9 mm. Separately, a larger 6.3 mm mandrel was dip-coated with a thin film of the stiffer MDX4-4210 using hexane as a solvent. After curing, the elastic tube was stretched over the stiffer film on the 6.3 mm mandrel, bonding the layers with uncured soft silicone. The resulting bilayer tube was then removed from the 6.3 mm mandrel. After removal, the bilayer tube recoiled to approximately 3.2 mm inner diameter (ID) and formed uniform longitudinal corrugations of the stiffer luminal film, creating a tube with wrinkled luminal topography in its resting state (Figure 1C). Grafts with smooth luminal surfaces were fabricated using a luminal film layer of the same stiffness as in the wrinkled grafts but without the size mismatch between the two mandrels. Wrinkle structure was confirmed by brightfield microscopy (Nikon, Ti Eclipse, Melville, NY, United States) (Figure 1D) and by confocal microscopy (Olympus, FV3000, Center Valley, PA, United States) (Figure 1E). Wrinkle sizes/wavelengths were varied by adjusting the ratio of the stiff MDX:hexane in the dip-coating step.

To measure graft compliance, graft segments were mounted on a continuous flow system. By regulating the outflow of the grafts, the pressure within the graft could be tuned. The graft diameter at each pressure was measured from still images of the graft.

### Pulsatile Flow Circuit

A pulsatile flow circuit (Harvard Apparatus, Holliston, MA, United States) generated pulsatile flows that mimic the flow conditions created by cardiac function. The pump was set to a frequency of 40 strokes/min with an injection volume of 5 mL (200 mL/min). Grafts were cyclically distended in this circuit for 1 h under physiologic pressures. A pressure transducer (Vernier, Beaverton, OR, United States) was placed in the mid graft to monitor pressure (Logger Lite Software, Vernier). Peak pressures were maintained between 100 and 120 mmHg. Pooled human platelets, obtained from the UPMC blood bank within 1 day of expiration, were activated with thrombin (10U/200 mL platelets; EMD Millipore, Burlington, MA, United States) immediately before being placed into the flow circuit. Each set of experiments was conducted with the same batch of platelets to control for batch to batch variability in platelet activity. In some experiments, grafts were constrained externally with a stiff tube (∼5 mm ID) to prevent distension under pulsatile flow. The graft distal to the constrained portion remained dynamic and could undergo cyclical distention in the same flow circuit, holding the flow volume and rate constant between the constrained and the dynamic portions of the graft. Both smooth and wrinkled grafts were tested in this circuit.

### Continuous Flow Circuit

New Era NE-9000 Peristaltic Pump (Scientific Instrument Services, Ringoes, NJ, United States) was used to create a continuous, non-pulsatile flow circuit. A dampener consisting of a 500 mL saline bag was placed into the circuit to eliminate pulsatility from the peristaltic pump. The flow distal to this dampener was non-pulsatile but maintained equal flow rates (200 mL/min) as in the pulsatile system. This experiment was also conducted with thrombin-activated pooled human platelets under continuous flow for 1 h.

### Graft Staining

After exposure to platelets in the flow circuits, grafts were fixed in 4% paraformaldehyde for 1 h and then treated with Wright stain (Polysciences, Inc., Warrington, PA, United States) per manufacturer instructions. Grafts were opened for *enface* imaging for adherent platelets.

### Graft Imaging and Image Acquisition

Graft segments were imaged using brightfield microscopy (Ti Eclipse, Nikon, Melville, NY, United States) and differential interference contrast (DIC) microscopy (Olympus FV100). Wrinkled grafts were also imaged by scanning electron microscopy (SEM; JEOL 9335, JEOL, Peabody, MA, United States) to evaluate the surface structure of the material. Brightfield images were used to quantify adherent platelets. DIC images were used to measure luminal film thickness.

### Optical Coherence Tomography (OCT)

C7 Dragonfly optical coherence tomography (OCT) system (Abbott, Saint Paul, MN, United States) was employed to image graft lumens under pulsatile flow. The OCT catheter was positioned in the midportion of the grafts and videos were obtained per manufacturer’s instructions. Imaging was performed using saline in the flow circuit to optimize imaging quality.

### Quantification of Platelet Deposition

ImageJ software (NIH, Bethesda, MD, United States) was used to quantify platelet deposition on the luminal surface of grafts. Images were converted to 8-bit for analysis, and threshold analysis was obtained on grafts for comparison. Ten high power fields (HPFs) were quantified for each graft, and the mean was reported for each graft. Three to five grafts were analyzed for each experimental group. Results are reported as mean ± standard error of the mean (SEM). Data were analyzed using Wilcoxon signed-rank test for pair wise comparisons and one-way ANOVA on ranks with Dunn’s test for multiple pairwise comparisons (SigmaStat, Systat Software, Inc., San Jose, CA, United States).

## Results

### Fabrication of Grafts With Wrinkled Luminal Topography

**FIGURE 2:**
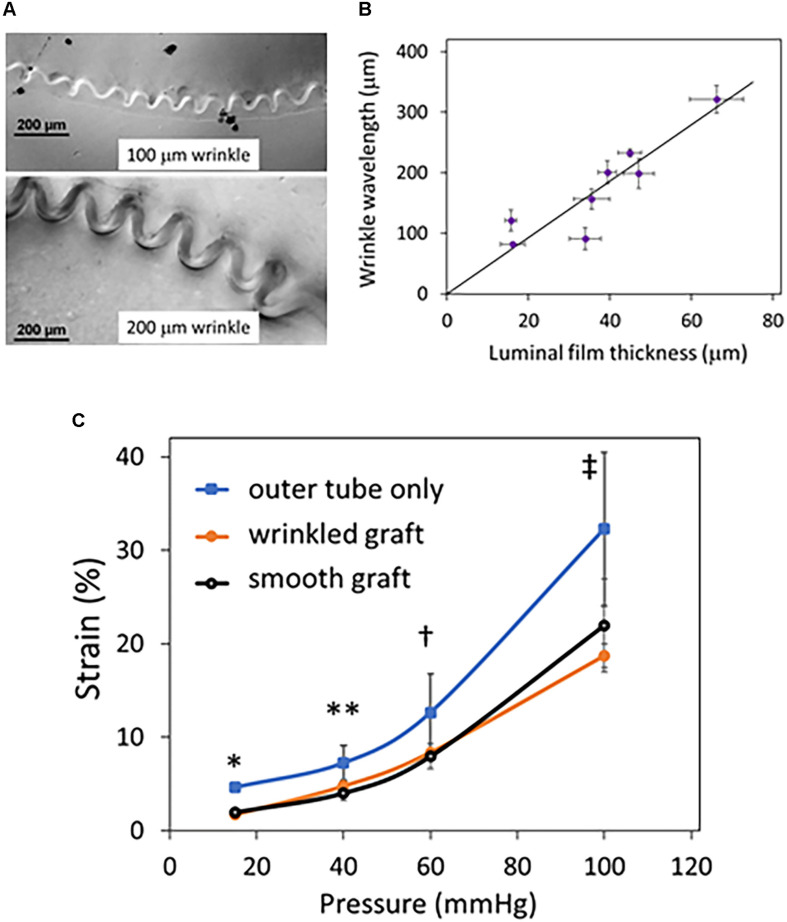
Effect of luminal film on graft wrinkle size and graft compliance. Grafts were fabricated with a thin stiff luminal film that determined the size of the luminal wrinkles when the outer, more elastic tube recoiled. **(A)** Sample cross-sectional differential interference contrast (DIC) microscopy images of wrinkled grafts are shown and focus on the luminal film layer. The thickness of the luminal film determines the size of the wrinkles with thicker films creating larger wrinkles. **(B)** The thickness of the luminal film and the wavelength were measured from DIC images of eight different grafts and plotted. The graph shows that wavelength increases proportionately with film thickness. Error bars are the standard deviations in wrinkle wavelength and luminal film thickness from several measurements taken along each individual graft. **(C)** Grafts with wrinkled and smooth luminal topography with the same luminal film thickness were tested for compliance. Inflation characteristics of these grafts were examined under continuous flow. The outer tube without the luminal film was evaluated for comparison only; no other experiment was performed with the outer tube. The % change in strain is plotted versus luminal pressure. The grafts with wrinkled and or smooth luminal film have similar inflation characteristics (P = NS at all pressures), whereas the tube has higher distensibility (*N* = 4 grafts/condition; ^∗^*P* < 0.001 between outer tube vs. smooth and wrinkled grafts; ^∗∗^*P* = 0.049, outer tube vs. wrinkled graft and *P* = 0.1 outer tube vs. smooth graft; ^†^*P* < 0.067 outer tube vs. wrinkled and smooth grafts; ‡ *P* = 0.04 outer vs. smooth grafts and *P* = 0.087 outer vs. wrinkled grafts).

Compliant bilayer silicone grafts with uniform wrinkled luminal topography were successfully fabricated (Figures 1C–E) by taking advantage of the strain mismatch between two materials of different stiffness (Figure 1B). By SEM, the nanoscale topography of these grafts was seen to be uniform and smooth (Figure 1E). Wrinkle wavelengths were varied by adjusting the thickness of the inner film (Figures 2A,B). Specific film thicknesses reproducibly generated wrinkle wavelengths ranging from 100 to 300 μm (Figure 2B). Smooth grafts were constructed by matching the diameter of the inner film to the inner diameter of the outer tube. Mechanical testing of these grafts demonstrated similar compliance between the wrinkled and the smooth grafts (Figure 2C) while the outer tube by itself is more compliant. The elastic properties of the bilayer graft were chosen to allow about 10–20% change in the graft diameter under distending pressures consistent with human systolic blood pressures (120–140 mmHg).

### Wrinkled Grafts Demonstrate Dynamic Luminal Topography Under Pulsatile Flow

**FIGURE 3:**
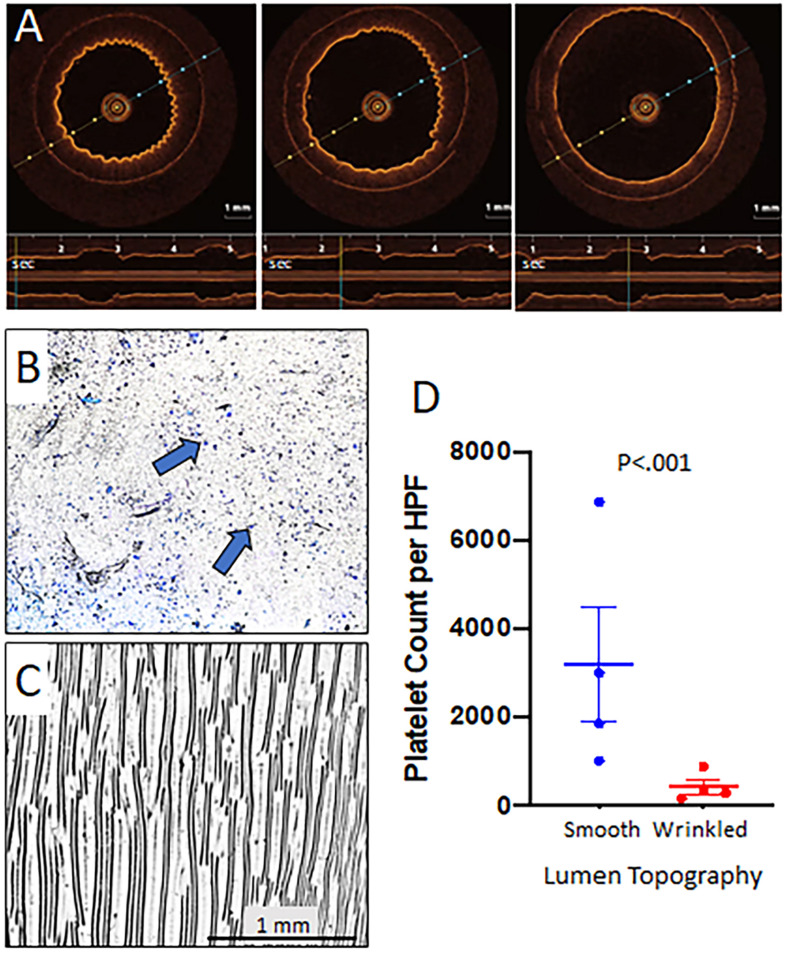
Dynamic topography of wrinkled grafts and effect on platelet adherence. Grafts with wrinkled luminal surfaces were placed in a pulsatile flow circuit with peak pressures of 140 mmHg. Representative OCT images reveal progressive flattening of the luminal surface wrinkles as luminal pressure increased (repeated with four different grafts). The timeline at the bottom of each image shows a pulse frequency of 36 per minute and the reproducible distention of the graft in that location with each pulse **(A)**. These experiments were performed using wrinkled grafts with ∼200 μm wavelength to allow better visualization on OCT to demonstrate dynamic topography. Smooth and wrinkled bilayer grafts were placed on a pulsatile flow pump with thrombin activated platelets. Peak pressures were set for 140 mmHg while the resting pressure was 60–80 mmHg to correlate with diastolic pressures *in vivo*. The pulse rate was set to 40 beats/minute. Grafts were removed at 1 h and stained with Wright stain to detect accumulated platelets. Platelet deposition per high powered field (HPF) was quantified using ImageJ software. Representative photomicrographs revealed that smooth grafts **(B)** exhibited significantly more adherent platelets (blue dots and arrows) as compared with wrinkled grafts (wrinkle wavelength ∼100 μm) **(C)**. Summary of the results for the experiment are shown graphically (*N* = 4 grafts/treatment group, 10 HPFs were quantified per graft) **(D)**. The comparison of platelet accumulation between smooth and wrinkled grafts was conducted using Wilcoxon signed Rank test.

Grafts were placed on a syringe pump for fidelity testing and tolerated distention to pressures of 140–180 mmHg without rupture and recoiled under lower pressures. In the pulsatile pump circuit, the luminal surface of wrinkled grafts flattened at the peak of pulsatile flow and returned to the wrinkled state during the low-pressure segment of the pulsatile cycle as imaged by OCT (Figure 3A and Supplementary Video 1). This cyclical flattening and wrinkling of the luminal surface confirmed the achievement of reproducible dynamic topography with properly tuned graft compliance. Grafts tolerated up to 14 days of pulsatile flow without disruption of the luminal topography or fatigue of the silicone tube (data not shown).

### Dynamic Luminal Topography Resists Platelet Adhesion

To examine the effect of luminal topography on platelet adherence, wrinkled and smooth grafts were placed on the pulsatile pump circuit with activated platelets for 1 h. Smooth surface grafts exhibited marked luminal platelet adhesion (3162 ± 418/HPF) (Figures 3B,D) while grafts with dynamic wrinkled luminal topography exhibited significantly fewer adherent platelets (401 ± 59/HPF; *P* < 0.001) (Figures 3C,D), representing an 86% reduction in platelet accumulation compared to smooth grafts.

### Role of Dynamic Topography in Antiplatelet Function

**FIGURE 4:**
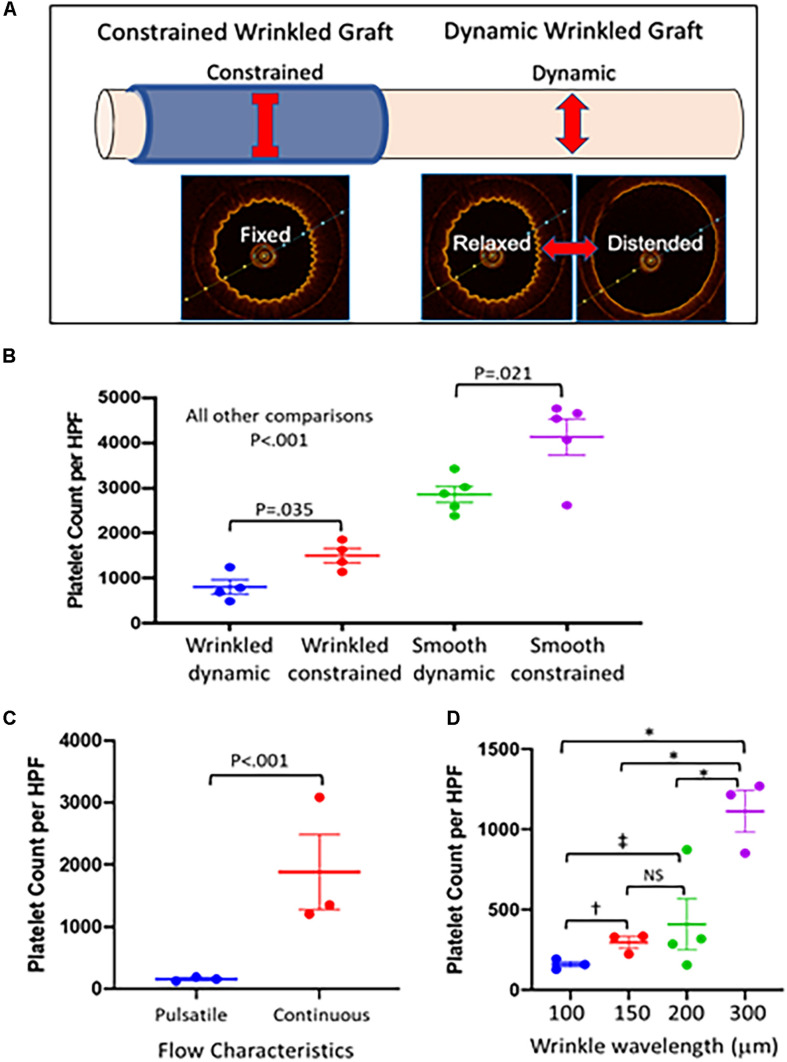
Effect of luminal surface behavior on platelet deposition. **(A)** To test the role of dynamic topography in resisting platelet adherence, a portion of the dynamic graft was externally constrained to prevent graft distension during pulsatile flow to maintain a fixed wrinkled luminal surface under pulsatile flow. The adjacent dynamic portion of graft continued to undergo distension during pulsatile flow and cycled between wrinkled and smooth luminal configurations. **(B)** Grafts were placed on pulsatile pump conditions with thrombin activated platelets for 1 h, stained with Wright stain, and platelet deposition per high powered field was quantified using Image J software. Externally constrained wrinkled grafts (*n* = 4–5 grafts/condition) demonstrated increased platelet deposition compared with dynamic wrinkled grafts (*P* < 0.035). Similarly, constrained smooth grafts also had increased platelet deposition compared to dynamic smooth grafts (*P* < 0.021). Dynamic wrinkled grafts were more resistant to platelet deposition than smooth dynamic grafts (*P* < 0.001). Constrained wrinkled grafts were also more resistant to platelet accumulation than constrained smooth grafts (*P* < 0.001). Comparisons were performed with one-way ANOVA with Dunn’s test. **(C)** Wrinkled grafts (∼100 μm wavelength, *n* = 3 grafts) were either placed on pulsatile (dynamic topography) or continuous (fixed topography) flow conditions for 1 h with activated platelets. Grafts undergoing pulsatile flow had significantly lower platelet adherence (*P* < 0.001) than those exposed to continuous flow. The comparison was performed with the Wilcoxon signed-rank test for pair wise comparisons. **(D)** Grafts with different wavelength wrinkles were placed under pulsatile flow with activated platelets. Comparisons were performed with one-way ANOVA with Dunn’s test for multiple comparison. In comparison with grafts with 300 μm wrinkles, those with smaller wrinkle sizes were significantly more resistant to platelet aggregation (*N* = 3–4 grafts/wavelength; ^∗^*P* < 0.001). Grafts with 100 μm wrinkles showed a trend toward a significant reduction in platelet adherence compared to grafts with 150 μm wrinkles (^†^*P* = 0.073) and significantly reduced platelet adherence versus grafts with 200 μm wrinkles (^‡^*P* = 0.005).

To evaluate the specific role of the oscillation of the luminal surface between wrinkled and flat configurations (dynamic topography) in preventing platelet adhesion, we externally constrained a portion of the graft to create static topography during pulsatile flow (Figure 4A). This allowed pulsatile flow to be maintained through the graft without inducing changes in topography. As detected by OCT imaging, the external constraint was not a perfect fit and did not completely eliminate pulsatile changes to the wrinkles but did reduce the changes in wrinkle amplitudes substantially (data not shown). The constrained sections of the wrinkled graft exhibited a doubling of platelet accumulation compared to the downstream unconstrained, dynamic segments of graft (Figure 4B). Constrained smooth grafts exhibited further increases in platelet accumulation compared with the dynamic segments of smooth grafts (Figure 4B), indicating that surface strain during cyclical distention itself can also reduce platelet adhesion irrespective of luminal topography (Shivapooja et al., 2013; Pocivavsek et al., 2019). Under either dynamic or constrained flows, the wrinkled grafts show significantly reduced platelet adherence compared to smooth grafts (*P* < 0.001 for wrinkled versus smooth grafts under both dynamic and constrained conditions).

### Wrinkled Grafts Exhibit Increased Platelet Adherence During Non-pulsatile Flow

A critical question is whether the dynamic nature of graft topography, i.e., the fact that the surface changes with pulsatile flow, is important to the graft’s ability to resist platelet adherence. Therefore, we examined grafts under non-pulsatile, continuous flow which did not alter graft topography. The flow rate was equivalent to the average flow rate in the pulsatile flow experiments, and a pressure of ∼100 mmHg was maintained. Wrinkled grafts placed in this continuous flow circuit with activated platelets exhibited > 90% increase in platelet deposition as compared to wrinkled grafts under pulsatile flow (Figure 4C). This experiment illustrated the importance of dynamic changes in graft topography in resisting platelet accumulation. It is noteworthy that the density of adhered platelets on the wrinkled grafts was similar between the continuous flow and constrained pulsatile flow experiments (Figures 4B,C). This supports the hypothesis that the mechanism of platelet de-adhesion is driven by the fluctuating surface topography and not by the characteristics of the fluid mechanics itself because pulsatile flow in the absence of dynamic topography or changes in luminal surface strain did not reduce platelet adhesion.

### Influence of Luminal Wrinkle Size on Antiplatelet Function

In previous experiments (Pocivavsek et al., 2019), the smallest wrinkle wavelength that we tested was 80 μm which had the strongest antiplatelet effect. In pig carotid arteries which are similar to human arteries in size and composition, the wrinkle sizes were smaller in the 20 μm range (Nguyen et al., 2020) (Figure 1A). To determine the impact of wrinkle size on the antiplatelet properties of the wrinkled grafts, we fabricated grafts with wrinkle wavelengths ranging from ∼100 to 300 μm. The size of the wrinkles in experiments in Sections “Fabrication of Grafts With Wrinkled Luminal Topography,” “Wrinkled Grafts Demonstrate Dynamic Luminal Topography Under Pulsatile Flow,” “Dynamic Luminal Topography Resists Platelet Adhesion,” “Role of Dynamic Topography in Antiplatelet Function,” and “Wrinkled Grafts Exhibit Increased Platelet Adherence During Non-pulsatile Flow” was ∼100 μm. Under pulsatile flow, grafts with 100 μm wrinkles exhibited the lowest levels of platelet accumulation compared to grafts with larger wrinkles (Figure 4D). Grafts with 300 μm wrinkles behaved very similar to smooth surface grafts.

## Discussion

While luminal corrugations or wrinkles are evident on arterial histology, no specific function has been attributed to these structures. The study of material interfaces, however, predicts a potential role for cyclical fluctuations between wrinkled and smooth topography in preventing material adherence to the surface (Shivapooja et al., 2013). Through computational analysis and experiential work, we have reported that dynamic wrinkling at an interface can exert strain on an adherent substance, resulting in detachment from that interface (Pocivavsek et al., 2018). We speculate that the unique macrostructure of the arterial lumen-blood interface may exist to shed adherent platelets and thrombus. This hypothesis has yet to be investigated but improved arterial modeling methodologies, such as the Ogden-Gasser-Holzapfel method, will help to provide some insight into the role of dynamic arterial topography (Nguyen et al., 2020).

Regardless of its role in native arteries, the concept of dynamic surface topography can be applied to cardiovascular devices to prevent the serious complication of platelet thrombus formation that can lead to catastrophic embolic events or vascular occlusion. The concept that dynamic topography can resist material adherence was reported by Pocivavsek et al. (2018). We fabricated elastic bilayer surfaces that possessed wrinkled surface topography in its resting state. When cyclically transitioned between wrinkled and smooth configurations, these grafts repelled platelets in comparison with static wrinkled surfaces or a smooth surfaces undergoing cyclic stretch (Pocivavsek et al., 2019). Further experiments were conducted by creating cylindrical tubes from these flat bilayers, orienting the wrinkles longitudinally along the inner surface (Pocivavsek et al., 2019). Cyclical inflation and deflation of these tubes created dynamic luminal topography which similarly resisted platelet deposition as compared to tubes with smooth luminal surfaces. These early studies were limited by the use of industrial grade materials to fabricate the bilayers, the suturing of the bilayers into cylinders that created a highly thrombogenic seam, and the use of a reciprocal action syringe pump to create to-and-fro flow to mimic the pulsatile blood flow. Despite these limitations, the findings provided proof of concept for the novel application of dynamic topography for surface cleansing and were the bases for the current investigation.

One of the most problematic areas of cardiovascular therapies is the susceptibility of vascular devices, namely vascular grafts, to platelet accumulation and thrombus formation. The limited success of surface modifications, such as heparin bonding (Devine et al., 2004; Bosiers et al., 2006; Neville et al., 2012; Uhl et al., 2017), continues to drive the ongoing search for better innovations in vascular graft technology. In this study, we extended our previous work by fabricating compliant tubular grafts with linearly oriented wrinkles using medical grade silicones (Figures 1B–E). The fabrication relied upon the differences in elastic properties of a stiff thin luminal layer and a more elastic thicker outer tube. The recoil of the outer tube created the reproducible wrinkling pattern of the larger diameter inner film, generating a tubular construct with a uniform, corrugated luminal surface at low pressures. This method of compression-induced wrinkling reproducibly created 100-micron scale topography on the luminal surface of a tube without the need for complex microfabrication techniques. This method also allowed tuning of wrinkle sizes by simple modification of luminal film thickness (Figures 2A,B). In a pulsatile flow circuit, these compliant grafts underwent cyclical distension/relaxation with reversible flattening/wrinkling of the luminal surface (Supplementary Video 1) which significantly reduced platelet adhesion compared to grafts with smooth luminal surfaces, reinforcing the ability of dynamic topography to resist platelet adhesion.

The wrinkled topography of the graft itself does not drive the antiplatelet effects. We previously showed that, under static conditions, the wrinkled surface increased platelet accumulation compared to a smooth surface due to the greater surface area of the wrinkles (Pocivavsek et al., 2019) as well as the greater thrombogenicity arising from the increased surface roughness created by the wrinkles (Gu et al., 2016). It is the cyclical change between wrinkled and flat surface conformations that drives the antiplatelet activity as evidenced by increased platelet adherence when wrinkled grafts were constrained to create static topography in the setting of pulsatile flow. This finding indicates that pulsatile flow itself does not impart antiplatelet effects but serves as the driver of dynamic topography that mediates the shedding of platelets. Further support for this was the persistence of platelet aggregates in grafts with smooth luminal surfaces where pulsatile graft behavior had much less impact. In our current experiments, the constrained wrinkled graft still exhibited less platelet adherence than the constrained smooth grafts (Figure 3C), contradicting our prior observations of the greater thrombogenicity of static wrinkled grafts (Pocivavsek et al., 2019). This may be due to the imperfect fit of the external constraint and the grafts continued to exhibit ongoing but reduced dynamic behavior which likely contributed to the reduced platelet adherence of the wrinkled grafts.

The critical importance of the dynamic fluctuation between smooth and wrinkled configurations in resisting platelet aggregation was further illustrated by experiments using a continuous flow circuit to eliminate pulsatile flow within the wrinkled grafts while maintaining flow rates, and pressures. Continuous flow increased platelet deposition in the wrinkled grafts by 94% compared to grafts exposed to pulsatile flow. These current experiments produced similar antiplatelet behavior to our prior work that utilized non-flow-based surface distention and offer strong evidence to support elasticity-based topographic surface renewal model (Pocivavsek et al., 2019). Constant deformation of the luminal surface forced delamination of activated platelets which can prevent the recruitment of circulating platelets (Patel et al., 2003; Dubois et al., 2007) and inflammatory cells that further accelerate thrombus formation and inflammatory activation. These findings also support our hypothesis that dynamic vascular topography may be an innate arterial defense against thrombosis.

Along with the dynamic component of surface topography, the wrinkle wavelength plays an important role in surface cleansing. Wavelengths of ∼100 μm achieved the lowest level of platelet accumulation as compared to grafts with larger wrinkles. Interestingly, pig arteries, which are comparable to human arteries, have luminal wrinkles with that are much small with an estimated wavelength of 20 μm (Nguyen et al., 2020). Unfortunately, testing of smaller wrinkles was not feasible due to the limitations of our graft fabrication method. However, the significant antiplatelet function achieved with grafts with 100 μm wrinkles, a wrinkle wavelength that is easily produced, suggests that this wrinkle size would be effective for clinical applications.

Compliance mismatch between a stiff prosthetic graft and the adjacent artery is a primary contributor to graft failure. Abbott et al. (1987) reported in 1987 a 50% failure rate of carotid artery autografts fixed with glutaraldehyde to generate a stiff graft. In partially fixed autografts that retained some arterial compliance, the patency rate was 100%. These findings and others (Greenwald and Berry, 2000) support the importance of compliance matching between the native artery and the bypass conduit. PTFE and PET vascular grafts are much stiffer than arteries (Tai et al., 2000), resulting in significant compliance mismatch at arterial anastomoses. Experimental grafts fabricated with polyurethane exhibited improved compliance properties and improved patency in animal models but did not perform well in humans (Ravi et al., 2009). The compliance of our silicone grafts offers a closer match to native arteries. This compliance matching itself may confer an antiplatelet function as supported by the reduced platelet adherence in smooth grafts undergoing pulsatile behavior. Graft failure secondary to compliance mismatch is due to the development of intimal hyperplasia at the distal anastomosis (Abbott et al., 1987; Greenwald and Berry, 2000). Therefore, grafts engineered to have compliance similar to native arteries and dynamic topography may demonstrate superior graft patency by both reducing platelet accumulation to prevent early graft thrombosis and reducing the mechanical and platelet-mediated stimuli for intimal hyperplasia that contribute to intermediate and long-term failure.

Endothelial cells are important to the homeostasis of blood vessels through the release of vasoprotective molecules such as nitric oxide and prostacyclin. Efforts to enhance EC growth or retention on prosthetic grafts to improve graft patency have not yielded much success (Seifalian et al., 2002). EC growth on these grafts is usually limited to 1–2 cm beyond the arterial anastomosis (Guidoin et al., 1993). While we have not evaluated the ability of our dynamic wrinkled grafts to support endothelial growth, the lack of porosity of the silicone film (Figure 1E) is a less favorable environment for endothelial growth and retention (Golden et al., 1990). In addition, dynamic topography may impair EC attachment, especially if the cells originate from circulating EC progenitor cells. However, we propose that dynamic topography will create an antithrombotic surface and will obviate the need for EC coverage.

Another perceived limitation of our graft is that it is not biodegradable and will be a permanent implant. An alternative view is the prosthetic nature of this graft may actually be an advantage. Unlike biodegradable tissue engineered vascular grafts where the balance between matrix degradation and cellular remodeling of the scaffold is difficult to achieve and the cost of producing and testing these grafts is likely prohibitive, dynamic prosthetic grafts can be manufactured at a fraction of the cost and the safety of the graft can be much more easily tested to allow for rapid translation to human use.

While our experiments used an *ex vivo* system to mimic *in vivo* pulsatile conditions, we acknowledge the limitations in accurately modeling *in vivo* flow conditions. Nonetheless, our pulsatile circuit offers conditions that cyclically distend the grafts with predictable flow characteristics to test platelet aggregation. The contribution of other circulating cells to thrombus propagation was also lacking in our model. Because the effects of dynamic topography are mechanical, we predict that the disruptive effect on surface fouling will be independent of cell type and cellular interactions. These *ex vivo* experiments do demonstrate our ability to fabricate compliant bilayer grafts with wrinkled luminal topography that possess mechanical properties similar to arteries, the grafts can oscillate between wrinkled and flat surface configurations under physiologic conditions of pulsatile flow, and that dynamic topography reduces platelet accumulation. Our future focus will be to test these grafts *in vivo* in large animals to evaluate graft behavior under true physiologic flow with all the blood components and the impact on thrombus formation over longer time intervals.

There are important implications of our findings for future technology with the most obvious being vascular graft innovation. While the ideal vascular conduit remains the autologous vein graft, innovations in prosthetic graft design may significantly improve their performance such that they can be viable alternatives for vein. This technology will likely be achievable at a fraction of the cost of the current efforts in tissue engineered vascular grafts. Other applications for dynamic topography include any blood contact surface that is prone to thrombosis such as dialysis circuits and in artificial heart design.

## Conclusion

Thrombosis is a significant contributor to the early failure of vascular grafts. Arteries possess a unique luminal structure that is corrugated. Cyclical dynamic fluctuation of a wrinkled surface to smooth reduced surface soiling, suggesting a potential role for arterial wrinkles. The incorporation of wrinkled topography onto the luminal surface of compliant vascular grafts that undergo cyclical changes in luminal topography reduced platelet accumulation. These findings support developing compliant prosthetic vascular conduits with dynamic topography and raises the fundamental question of whether arterial wrinkles may serve a similar anti-thrombotic function in nature which deserves further investigation.

## Data Availability Statement

The raw data supporting the conclusions of this article will be made available by the authors, without undue reservation.

## Author Contributions

NN, JP, YG, KS, and NP fabricated the grafts and performed the experiments. NN, LP, JP, YG, KS, and NP analyzed data and statistical analysis. NN, LP, RM, SV, and ET designed experiments and concepts. NN, LP, SV, and ET prepared and edited the manuscript. JP, YG, KS, NP, and RM reviewed and edited manuscript. LP, SV, and ET obtained funding for the studies. All authors contributed to the article and approved the submitted version.

## Conflict of Interest

LP, JP, SV, and ET have filed for intellectual property based on this technology and are working toward commercialization. The remaining authors declare that the research was conducted in the absence of any commercial or financial relationships that could be construed as a potential conflict of interest.

## References

[B1] Abbott W. M. Megerman J. Hasson J. E. L’Italien G. Warnock D. F. (1987). Effect of compliance mismatch on vascular graft patency. *J. Vasc. Surg.* 5 376–382. 10.1016/0741-5214(87)90148-0 3102762

[B2] Albers M. Romiti M. Brochado-Neto F. C. Pereira C. A. (2005). Meta-analysis of alternate autologous vein bypass grafts to infrapopliteal arteries. *J. Vasc. Surg.* 42 449–455. 16171586 10.1016/j.jvs.2005.05.031

[B3] Allen H. G. (1969). *Analysis and Design of Structural Sandwich Panels*, 1st Edn New York, NY: Pergamon Press.

[B4] Bennett M. R. Sinha S. Owens G. K. (2016). Vascular Smooth muscle cells in atherosclerosis. *Circ. Res.* 118 692–702. 26892967 10.1161/CIRCRESAHA.115.306361 PMC4762053

[B5] Bhushan B. (2009). Biomimetics: lessons from nature–an overview. *Philos. Trans. A Math. Phys. Eng. Sci.* 367 1445–1486. 10.1098/rsta.2009.0011 19324719

[B6] Biran R. Pond D. (2017). Heparin coatings for improving blood compatibility of medical devices. *Adv. Drug. Deliv. Rev.* 112 12–23. 10.1016/j.addr.2016.12.002 28042080

[B7] Bixler G. D. Bhushan B. (2012). Biofouling: lessons from nature. *Philos. Trans. A Math. Phys. Eng. Sci.* 370 2381–2417. 10.1098/rsta.2011.0502 22509063

[B8] Bosiers M. Deloose K. Verbist J. Schroe H. Lauwers G. Lansink W. (2006). Heparin-bonded expanded polytetrafluoroethylene vascular graft for femoropopliteal and femorocrural bypass grafting: 1-year results. *J. Vasc. Surg.* 43 313–318. discussion 318–319. 10.1016/j.jvs.2005.10.037 16476607

[B9] Cerda E. (2005). Mechanics of scars. *J. Biomech.* 38 1598–1603. 10.1016/j.jbiomech.2004.07.026 15958216

[B10] Devine C. McCollum C. North West Femoro-Popliteal Trial Participants (2004). Heparin-bonded Dacron or polytetrafluorethylene for femoropopliteal bypass: five-year results of a prospective randomized multicenter clinical trial. *J. Vasc. Surg.* 40 924–931. 10.1016/j.jvs.2004.08.033 15557906

[B11] Dubois C. Panicot-Dubois L. Gainor J. F. Furie B. C. Furie B. (2007). Thrombin-initiated platelet activation in vivo is vWF independent during thrombus formation in a laser injury model. *J. Clin. Invest.* 117 953–960. 10.1172/jci30537 17380206 PMC1821068

[B12] Furie B. Furie B. C. (2008). Mechanisms of thrombus formation. *N. Engl. J. Med.* 359 938–949. 10.1056/nejmra0801082 18753650

[B13] Genzer J. Groenewold J. (2006). Soft matter with hard skin: from skin wrinkles to templating and material characterization. *Soft. Matter.* 2 310–323. 10.1039/b516741h 32646128

[B14] Golden M. A. Hanson S. R. Kirkman T. R. Schneider P. A. Clowes A. W. (1990). Healing of polytetrafluoroethylene arterial grafts is influenced by graft porosity. *J. Vasc. Surg.* 11 838–844. discussion 845. 10.1067/mva.1990.18047 2359196

[B15] Greenwald S. E. Berry C. L. (2000). Improving vascular grafts: the importance of mechanical and haemodynamic properties. *J. Pathol.* 190 292–299. 10.1002/(sici)1096-9896(200002)190:3<292::aid-path528>3.0.co;2-s 10685063

[B16] Gu X. Mao Z. Ye S. H. Koo Y. Yun Y. Tiasha T. R. (2016). Biodegradable, elastomeric coatings with controlled anti-proliferative agent release for magnesium-based cardiovascular stents. *Colloids Surf. B Biointerfaces* 144 170–179. 10.1016/j.colsurfb.2016.03.086 27085049

[B17] Guidoin R. Chakfe N. Maurel S. How T. Batt M. Marois M. (1993). Expanded polytetrafluoroethylene arterial prostheses in humans: histopathological study of 298 surgically excised grafts. *Biomaterials* 14 678–693. 10.1016/0142-9612(93)90067-c 8399965

[B18] Hasebe T. Shimada A. Suzuki T. Matsuoka Y. Saito T. Yohena S. (2006). Fluorinated diamond-like carbon as antithrombogenic coating for blood-contacting devices. *J. Biomed. Mater. Res. A.* 76 86–94. 10.1002/jbm.a.30512 16138324

[B19] Ignarro L. J. Buga G. M. Wood K. S. Byrns R. E. Chaudhuri G. (1987). Endothelium-derived relaxing factor produced and released from artery and vein is nitric oxide. *Proc. Natl. Acad. Sci. U.S.A.* 84 9265–9269. 10.1073/pnas.84.24.9265 2827174 PMC299734

[B20] Jordan S. W. Chaikof E. L. (2007). Novel thromboresistant materials. *J. Vasc. Surg.* 45(Suppl. A), A104–A115. 17544031 10.1016/j.jvs.2007.02.048

[B21] Koh L. B. Rodriguez I. Venkatraman S. S. (2010). The effect of topography of polymer surfaces on platelet adhesion. *Biomaterials* 31 1533–1545. 10.1016/j.biomaterials.2009.11.022 19945746

[B22] Lavery K. S. Rhodes C. McGraw A. Eppihimer M. J. (2017). Anti-thrombotic technologies for medical devices. *Adv. Drug. Deliv. Rev.* 112 2–11. 10.1016/j.addr.2016.07.008 27496703

[B23] Li D. Zheng Q. Wang Y. Chen H. (2014). Combining surface topography with polymer chemistry: exploring new interfacial biological phenomena. *Polym. Chem.* 5 14–24. 10.1039/c3py00739a

[B24] Naoum J. A. Elias J. A. (2012). Bypass surgery in limb salvage: polytetrafluoroethylene prosthetic bypass. *Method. Debakey Cardiovasc. J,.* 8 43–46. 10.14797/mdcj-8-4-43 23342188 PMC3549650

[B25] Neville R. F. Capone A. Amdur R. Lidsky M. Babrowicz J. Sidawy A. N. (2012). A comparison of tibial artery bypass performed with heparin-bonded expanded polytetrafluoroethylene and great saphenous vein to treat critical limb ischemia. *J. Vasc. Surg.* 56 1008–1014. 10.1016/j.jvs.2012.03.020 22677009

[B26] Nguyen N. Nath N. Deseri L. Tzeng E. Velankar S. S. Pocivavsek L. (2020). Wrinkling instabilities for biologically relevant fiber-reinforced composite materials with a case study of neo-hookean/ogden-gasser-holzapfel bilayer. *Biomech. Model Mechanobiol.* 10.1007/s10237-020-01345-0 32535739 PMC7920575

[B27] Palmer R. M. Ferrige A. G. Moncada S. (1987). Nitric oxide release accounts for the biological activity of endothelium-derived relaxing factor. *Nature* 327 524–526. 10.1038/327524a0 3495737

[B28] Patel D. Vaananen H. Jirouskova M. Hoffmann T. Bodian C. Coller B. S. (2003). Dynamics of GPIIb/IIIa-mediated platelet-platelet interactions in platelet adhesion/thrombus formation on collagen in vitro as revealed by videomicroscopy. *Blood* 101 929–936. 10.1182/blood.v101.3.929 12529292

[B29] Pocivavsek L. Dellsy R. Kern A. Johnson S. Lin B. Lee K. Y. (2008). Stress and fold localization in thin elastic membranes. *Science* 320 912–916. 10.1126/science.1154069 18487188

[B30] Pocivavsek L. Leahy B. Holten-Andersen N. Lin B. Lee K. Y. C. Cerda E. (2009). Geometric tools for complex interfaces: from lung surfactant to the mussel byssus. *Soft. Matter.* 5 1963–1968. 10.1039/b817513f

[B31] Pocivavsek L. Pugar J. O’Dea R. Ye S.-H. Wagner W. Tzeng E. (2018). Topography-driven surface renewal. *Nat. Phys.* 14 948–953. 10.1038/s41567-018-0193-x PMC11271749 39055780

[B32] Pocivavsek L. Ye S. H. Pugar J. Tzeng E. Cerda E. Velankar S. (2019). Active wrinkles to drive self-cleaning: a strategy for anti-thrombotic surfaces for vascular grafts. *Biomaterials* 192 226–234. 10.1016/j.biomaterials.2018.11.005 30458358 PMC7248685

[B33] Quinones-Baldrich W. J. Prego A. A. Ucelay-Gomez R. Freischlag J. A. Ahn S. S. Baker J. D. (1992). Long-term results of infrainguinal revascularization with polytetrafluoroethylene: a ten-year experience. *J. Vasc. Surg.* 16 209–217. 10.1016/0741-5214(92)90109-l 1495144

[B34] Ravi S. Qu Z. Chaikof E. L. (2009). Polymeric materials for tissue engineering of arterial substitutes. *Vascular* 17(Suppl. 1), S45–S54. 19426609 10.2310/6670.2008.00084 PMC2714487

[B35] Russell T. P. (2002). Surface-responsive materials. *Science* 297 964–967. 10.1126/science.1075997 12169722

[B36] Seifalian A. M. Tiwari A. Hamilton G. Salacinski H. J. (2002). Improving the clinical patency of prosthetic vascular and coronary bypass grafts: the role of seeding and tissue engineering. *Artif. Organs.* 26 307–320. 10.1046/j.1525-1594.2002.06841.x 11952502

[B37] Shivapooja P. Wang Q. Orihuela B. Rittschof D. Lopez G. P. Zhao X. (2013). Bioinspired surfaces with dynamic topography for active control of biofouling. *Adv. Mater.* 25 1430–1434. 10.1002/adma.201203374 23292960

[B38] Svendsen E. Tindall A. R. (1988). The internal elastic membrane and intimal folds in arteries: important but neglected structures? *Acta Physiol. Scand. Suppl*. 572 1–71. 3232528

[B39] Tai N. R. Salacinski H. J. Edwards A. Hamilton G. Seifalian A. M. (2000). Compliance properties of conduits used in vascular reconstruction. *Br. J. Surg.* 87 1516–1524. 10.1046/j.1365-2168.2000.01566.x 11091239

[B40] Takagi H. Goto S. N. Matsui M. Manabe H. Umemoto T. (2010). A contemporary meta-analysis of Dacron versus polytetrafluoroethylene grafts for femoropopliteal bypass grafting. *J. Vasc. Surg.* 52 232–236. 10.1016/j.jvs.2010.02.010 20471778

[B41] Twine C. P. McLain A. D. (2010). Graft type for femoro-popliteal bypass surgery. *Cochrane Database Syst. Rev.* 5:CD001487. 10.1002/14651858.CD001487.pub2 20464717

[B42] Uhl C. Grosch C. Hock C. Topel I. Steinbauer M. (2017). Comparison of long-term outcomes of heparin bonded polytetrafluoroethylene and autologous vein below knee femoropopliteal bypasses in patients with critical limb ischaemia. *Eur. J. Vasc. Endovasc. Surg.* 54 203–211. 10.1016/j.ejvs.2017.05.001 28587797

[B43] Uttayarat P. Perets A. Li M. Pimton P. Stachelek S. J. Alferiev I. (2010). Micropatterning of three-dimensional electrospun polyurethane vascular grafts. *Acta Biomater.* 6 4229–4237. 10.1016/j.actbio.2010.06.008 20601235

[B44] Weber N. Wendel H. P. Ziemer G. (2002). Hemocompatibility of heparin-coated surfaces and the role of selective plasma protein adsorption. *Biomaterials* 23 429–439. 10.1016/s0142-9612(01)00122-3 11761163

[B45] Xu L. C. Siedlecki C. A. (2017). Protein adsorption, platelet adhesion, and bacterial adhesion to polyethylene-glycol-textured polyurethane biomaterial surfaces. *J. Biomed. Mater. Res. B Appl. Biomater.* 105 668–678. 10.1002/jbm.b.33592 26669615

[B46] Yang S. Khare K. Lin P. C. (2010). Harnessing surface wrinkle patterns in soft matter. *Adv. Funct. Mater.* 20 2550–2564. 10.1002/adfm.201000034

